# Targeting the IL-6 Dependent Phenotype Can Identify Novel Therapies for Cholangiocarcinoma

**DOI:** 10.1371/journal.pone.0015195

**Published:** 2010-12-16

**Authors:** Chiara Braconi, Erica Swenson, Takayuki Kogure, Nianyuan Huang, Tushar Patel

**Affiliations:** Department of Internal Medicine, College of Medicine, Ohio State University Comprehensive Cancer Center, Ohio State University, Columbus, Ohio, United States of America; National Cancer Institute, National Institutes of Health, United States of America

## Abstract

**Background:**

The need for new therapies for cholangiocarcinoma is highlighted by their poor prognosis and refractoriness to chemotherapy. Increased production of Interleukin-6 promotes cholangiocarcinoma growth and contributes to chemoresistance by activating cell survival mechanisms. We sought to identify biologically active compounds capable of ameliorating the phenotypic effects of IL-6 expression and to explore their potential therapeutic use for cholangiocarcinoma.

**Methodology:**

A genomic signature associated with Interleukin-6 expression in Mz-ChA-1 human malignant cholangiocytes was derived. Computational bioinformatics analysis was performed to identify compounds that induced inverse gene changes to the signature. The effect of these compounds on cholangiocarcinoma growth was then experimentally verified *in vitro* and *in vivo*. Interactions with other therapeutic agents were evaluated using median effects analysis.

**Principal Findings:**

A group of structurally related compounds, nitrendipine, nifedipine and felodipine was identified. All three compounds were cytotoxic to Mz-ChA-1 cells with an IC50 for felodipine of 26 µM, nitrendipine, 44 µM and nifedipine, 15 µM. Similar results were observed in KMCH-1, CC-LP-1 and TFK-1 cholangiocarcinoma cell lines. At a fractional effect of 0.5, all three agents were synergistic with either camptothecin or gemcitabine in Mz-ChA-1 cells *in vitro*. Co-administration of felodipine and gemcitabine decreased the growth of Mz-ChA-1 cell xenografts in nude athymic mice.

**Conclusions:**

Computational bioinformatics analysis of phenotype-based genomic expression can be used to identify therapeutic agents. Using this drug discovery approach based on targeting a defined tumor associated phenotype, we identified compounds with the potential for therapeutic use in cholangiocarcinoma.

## Introduction

Malignancies arising from the biliary tract, or cholangiocarcinomas, are uncommon tumors for which effective therapies are lacking [1], [2]. The incidence of intrahepatic cholangiocarcinomas has been noted to be increasing worldwide [3]–[6]. Unless surgical resection is possible, the prognosis is quite poor, and the tumors are characteristically refractory to conventional chemotherapy [7]. While gemcitabine or 5-fluorouracil have been used in clinical practice, response rates are poor and the prognosis is dismal. Several agents are currently being evaluated as single agents for cholangiocarcinoma including capecitabine and erlotinib [8], [9]. Although there has been interest in the evaluation of several of the currently available targeted therapies for use in cholangiocarcinoma, the rational use of these agents has been hindered by the paucity of knowledge of cellular mechanisms involved in cholangiocarcinoma growth or response to therapy [10], [11]. Consequently there remains a desperate need for more effective therapies, or for approaches that can improve therapeutic responses to treatment of this cancer.

Cholangiocarcinomas often arise in the setting of chronic biliary tract inflammation. The inflammation-associated cytokine Interleukin-6 (IL-6) is increased in the biliary tract and in the systemic circulation in patients with cholangiocarcinoma [12]–[15]. Autocrine expression of IL-6 has been described in cholangiocarcinoma cells, and IL-6 can promote the growth of malignant cholangiocytes *in vitro*. In recent studies, we have evaluated the role of IL-6 in growth regulation and chemotherapeutic responses in cholangiocarcinoma [16]–[19]. Furthermore, we have characterized phenotypic changes in response to enforced expression of IL-6 in tumor growth and resistance to chemotherapy [18]. These observations support the strategy of targeting the cellular effects of IL-6 for the treatment of cholangiocarcinoma.

To identify new targets for therapeutic intervention in cholangiocarcinoma, we postulated that a systematic approach based on ameliorating the phenotypic changes associated with IL-6 expression would be useful for identifying potential therapeutic agents. Our strategy consisted of deriving gene expression signatures that are associated with these phenotypic changes followed by computational bioinformatics analysis to identify biological active compounds capable of ameliorating these genetic changes. Candidate agents identified by this approach were then evaluated for their potential use in the treatment of cholangiocarcinoma.

## Results

### Derivation of a genomic signature of IL-6 expression

We began by first identifying a genomic signature that is associated with IL-6 over-expression. For these studies, we performed analysis in Mz-ChA-1 cells that were stably transfected with full-length IL-6 and which results in elevated basal IL-6 production, promotion of cell survival and resistance to chemotherapy and enhancement of tumor cell xenograft growth *in vivo* [18]. Whole genome analysis was performed using Affymetrix U133 Plus 2.0 arrays. A genomic signature consisting of 43 differentially expressed genes was generated by analyzing genes that were consistently present, and significantly altered in expression in IL-6 over-expressing cells compared to controls. This gene set comprised of 13 genes with increased expression and 30 genes with decreased expression. Of note, most genes in this signature gene set are not reported direct targets of IL-6 (Table S1) or genes that are implicated in tumor cell growth or survival. Nevertheless, we postulated that this would be a useful signature of the phenotype induced by IL-6 over-expression which has been experimentally shown to increase tumor growth in vivo.

### Identification of candidate compounds

**Figure 1:**
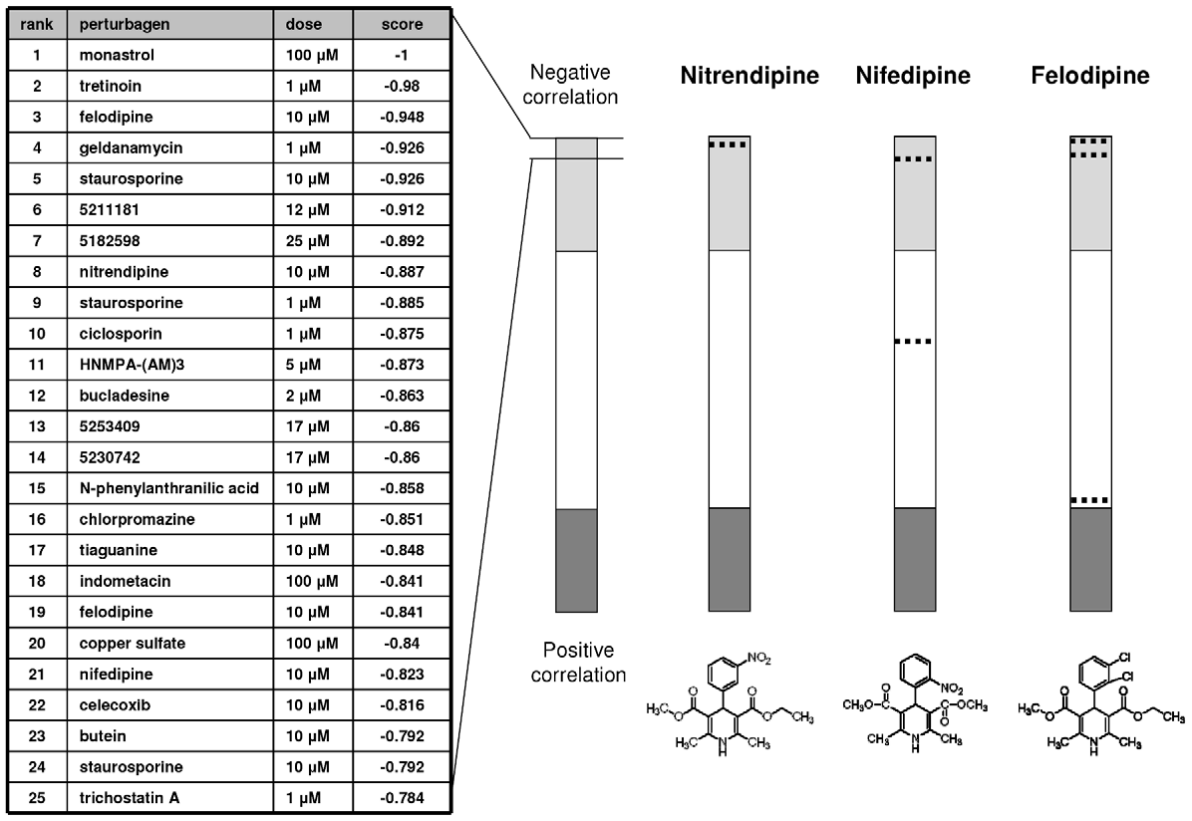
Computational bioinformatic analysis. The phenotype-based signature associated with IL-6 expression was correlated with gene expression profiles associated with diverse small molecule perturbagens using the Connectivity Map dataset. A "connectivity score" for each perturbagen was calculated based on the Kolmogorov-Smirnov statistic for both up- or down-regulated genes [35]. A negative score correlates inversely with the query gene signature. Results are depicted in rank order of connectivity scores ranging from −1 to +1. The light gray shaded bars represent perturbagens with a significantly negative correlation, whereas the dark gray bars at the bottom represent those with a significantly positive correlation. The top twenty-five instances with a highly significant (*p*<0.01) inverse correlation are listed in the left panel. These included three structurally similar compounds, nifendipine, nitrendipine and felodipine. Individual instances for each are depicted in the bars on the right as dotted lines. The mean connectivity scores for all instances of nifedipine, nitrendipine and felodipine were −0.412, −0.887 and −0.596 respectively indicating a significant negative correlation with the query signature.

In order to identify candidate small molecules capable to targeting the IL-6 associated phenotype, we performed computational bioinformatics analysis of the derived gene signature using the Connectivity Map. We postulated that compounds associated with genomic profiles that countered those associated with the IL-6 phenotype could provide useful leads for further study as therapeutic agents capable of ameliorating this phenotype. Thus we sought compounds with genomic profiles that had a negative correlation to our query signature. A search against 453 instances representing 164 bioactive small molecules identified several compounds which exhibited strong negative correlation to the query signature (Figure 1). These included several compounds which have been studied as cytotoxic drugs validating the use of this approach to identify meaningful candidates for further study. We have recently used this approach to identify candidate agents that can modulate the invasive cell phenotype in hepatocellular cancers [20]. The perturbagens from the Connectivity Map query were analyzed according to their permutated results and *p*-value, correlation scores, and potential for therapeutic use (Figure 1). The correlation scores reflect the similarity between the gene expression profiles and those caused by the phenotype of interest. A high negative correlation predicts that the agents could modulate the phenotype, but do not indicate their potency in doing so. Monastrol and Tretinoin emerged as the perturbagens with the highest negative correlation. Their anticancer properties are recognized, supporting the value of this approach [21]–[23]. Three structurally related compounds, felodipine, nifendipine and nitrendipine were identified suggesting a potential anticancer activity for this class of perturbagens. These compounds are functionally similar members of a class of clinically used agents with calcium channel blocker activity. Their extensive use in other disease and their known safety profile made them attractive for further study. Moreover, the activity of these agents in cancer remains unexplored. All three compounds had a mean connectivity score indicating a negative correlation with the query signature: nifedipine (-0.412), nitrendipine (-0.887), and felodipine (-0.596). We next interrogated an expanded version of the connectivity-map database (version 2), which includes 7056 genomic profiles representing 6100 individual treatment instances with 1309 bioactive small molecules. The three candidate agents retained their similar and highly significant negative scores in this broader analysis (Table 1). Interestingly two other calcium channel blockers, verapamil and diltiazem, also showed negative scores supporting the hypothesis that functional effects common to all drugs may be useful in reverting the systemic effects of IL-6 over-expression in cholangiocarcinoma.

**Table 1:** Analysis of the query gene signature using the Connectivity-map 2.0.

	Nitrendipine	Felodipine	Nifedipine	Verapamil	Diltiazem
Highest score	−0.88	−0.61	−0.58	−0.80	−0.55
Mean score	−0.5	−0.4	−0.2	−0.38	−0.38
Enriched score	−0.58	−0.51	−0.30	−0.47	−0.56
*P* value	0.03	0.03	0.49	0.09	0.046

The highest connectivity score recorded, arithmetic mean of the scores for all the instances in the database, measure of enrichment of those instances related to each agent and a *p*-value for the enrichment are listed.

### Candidate agents are cytotoxic to cholangiocarcinoma cells

**Figure 2:**
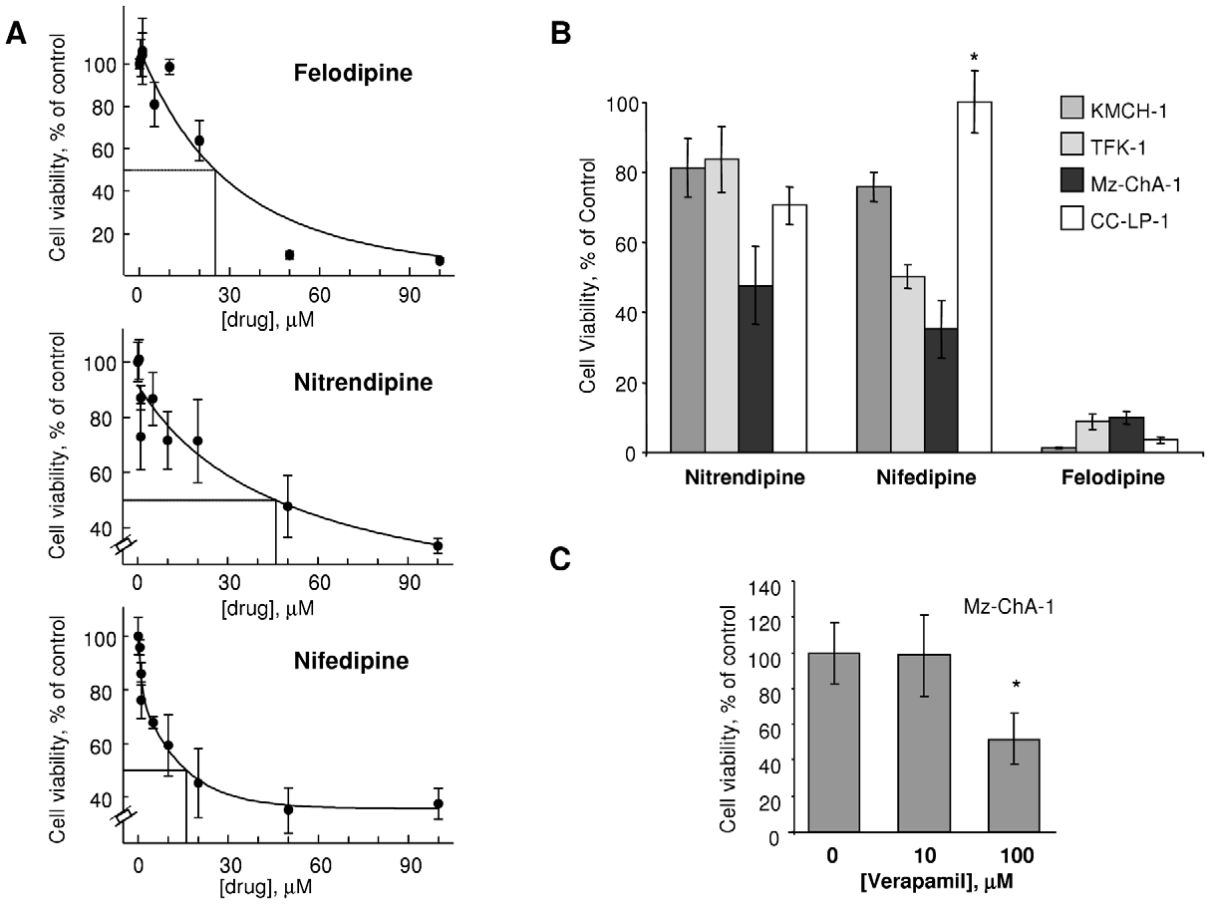
Cytotoxicity of candidate agents to cholangiocarcinoma cells. Panel A: Mz-ChA-1 cells were seeded into 96-well plates at a cell density of 5,000 cells per well in media with 5% FBS at 37°C with 5% CO2. After incubation overnight felodipine, nitrendipine, or nifedipine were added at the concentrations indicated, and cell viability assessed after 72 hours using a metabolic assay (CellTiter 96 AQ, Promega Corp., Madison, WI). Cytotoxicity as a percent of control is plotted against the concentration for each. The data represents the mean and standard deviation of five separate determinations. All three compounds decreased cell viability in a concentration-dependent manner, with an IC50 for felodipine of 26±5 µM, nitrendipine, 44±10 µM and nifedipine, 15±4 µM. Panel B: KMCH-1, CCLP-1, Mz-ChA-1 and TFK-1 cholangiocarcinoma cells were incubated with the candidate agents at a concentration of 50 µM. Cell viability was assessed after 72 hrs using a viable cell assay. A significant reduction in cell viability was observed with each agent and in each cell type, except as indicated (N:5* *p*>0.05 relative to controls). Panel C: Mz-ChA-1 cells were incubated with verapamil at the indicated concentrations or diluent control and cell viability assessed after 72 hours using a metabolic cell viability assay (N:7 * *p*<0.05 relative to control).

**Figure 3:**
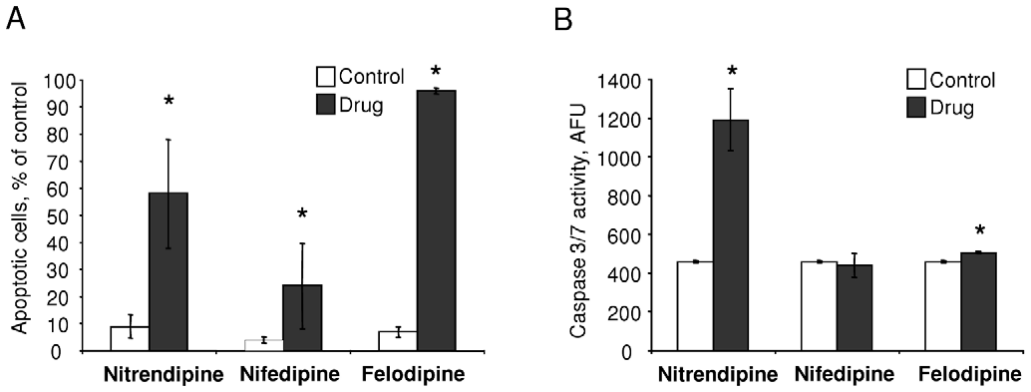
Candidate agents induce apoptosis in malignant cholangiocytes. Apoptosis was assessed morphologically and biochemically in Mz-ChA-1 cells incubated with felodipine (20 µM), nitrendipine (30 µM), or nifedipine (20 µM) for 24 hrs. Panel A: The number of cells with nuclear morphological features of apoptosis was counted using fluorescence microscopy after staining with DAPI, and is expressed as a percentage of all cells counted in several high power fields. Results are from three separate experiments. * p<0.05 relative to controls. Panel B: Caspase 3/7 activation was assessed using a luminometric assay (Caspase-Glo 3/7 Assay, Promega Corp., Madison WI). Compared to controls, significantly higher caspase 3/7 activation was observed with felodipine or nitrendipine. Results are from four separate experiments. * p<0.05 relative to controls.

To evaluate the potential of this group of drug candidates as therapeutic agents for cholangiocarcinoma, we began by evaluating their cytotoxic potential in IL-6 producing cholangiocarcinoma cell lines. All three compounds decreased Mz-ChA-1 cell viability in a concentration-dependent manner. The IC50 for felodipine was 26±5 µM, for nitrendipine, 44±10 µM and for nifedipine, 15±4 µM at 72 hours (Figure 2A). Similar analyses were also performed in other cell lines, with very similar results observed in KMCH, CC-LP-1 and TFK-1 cholangiocarcinoma cells (Figure 2B). Felodipine was the most effective in all the cell lines, in accordance with the multiple negative scores observed for this drug in the c-map analysis. Of note, similar IC50 values were observed in hepatocellular cancer cell lines (Figure S1). Hepatocarcinognesis has been recently proven to be driven by IL-6, supporting our findings [24], [25]. We also investigated the cytotoxic potential of verapamil, a structurally unrelated calcium channel blocker and observed a 48% reduction in Mz-ChA-1 cell viability after 72 hours (Figure 2C). Next, we assessed the effect of felodipine, nitrendipine and nifedipine on cell apoptosis. First we assessed nuclear morphological changes of apoptosis in cells during incubation with the candidate agents. All agents had a dramatic effect in inducing apoptosis with felodipine having the greatest effect (Figure 3A). To verify these observations, we also quantitated activation of caspases, a biochemical feature of apoptosis, and detected an increase in caspase 3/7 with nitrendipine and felodipine (Figure 3B). Thus, cytotoxicity of these agents likely involves induction of apoptosis.

### Role of calcium transport in cytotoxicity

To evaluate the potential role of IL-6 over-expression on cellular processes involved in calcium transport, we evaluated the mRNA expression of calcium channels, calcium pumps and calcium exchangers in Mz-IL-6 cells relative to that in parental Mz-ChA-1 cells (Table S2). Other than a 1.8-fold increase in expression of the calcium channel, voltage dependent, gamma subunit 6 mRNA (CACNG6), no significant changes in expression (<0.67-fold or >1.5-fold) were observed for any other calcium channels or pumps compared to control cells. To investigate the role of CACNG6 the effect of siRNA mediated down-regulation of CACNG6 was determined. Inhibition of CACNG6 reduced cell viability by 30% in Mz-IL-6 cells. Moreover, cytotoxicity of felodipine was enhanced in cells treated with siRNA to CANG6 in compared with siRNA control (Figure S2). In order to assess the effect of extracellular calcium on cytotoxicity, cells were incubated in calcium-free media for 24 hours, prior to addition of felodipine. However, no significant differences were noted between cytotoxicity after 24 hours in the presence or absence of extracellular calcium. These observations suggest the possibility that mechanisms other than modulation of calcium transport could contribute to the observed cytotoxicity of these agents.

### Interactions with chemotherapeutic agents

**Figure 4:**
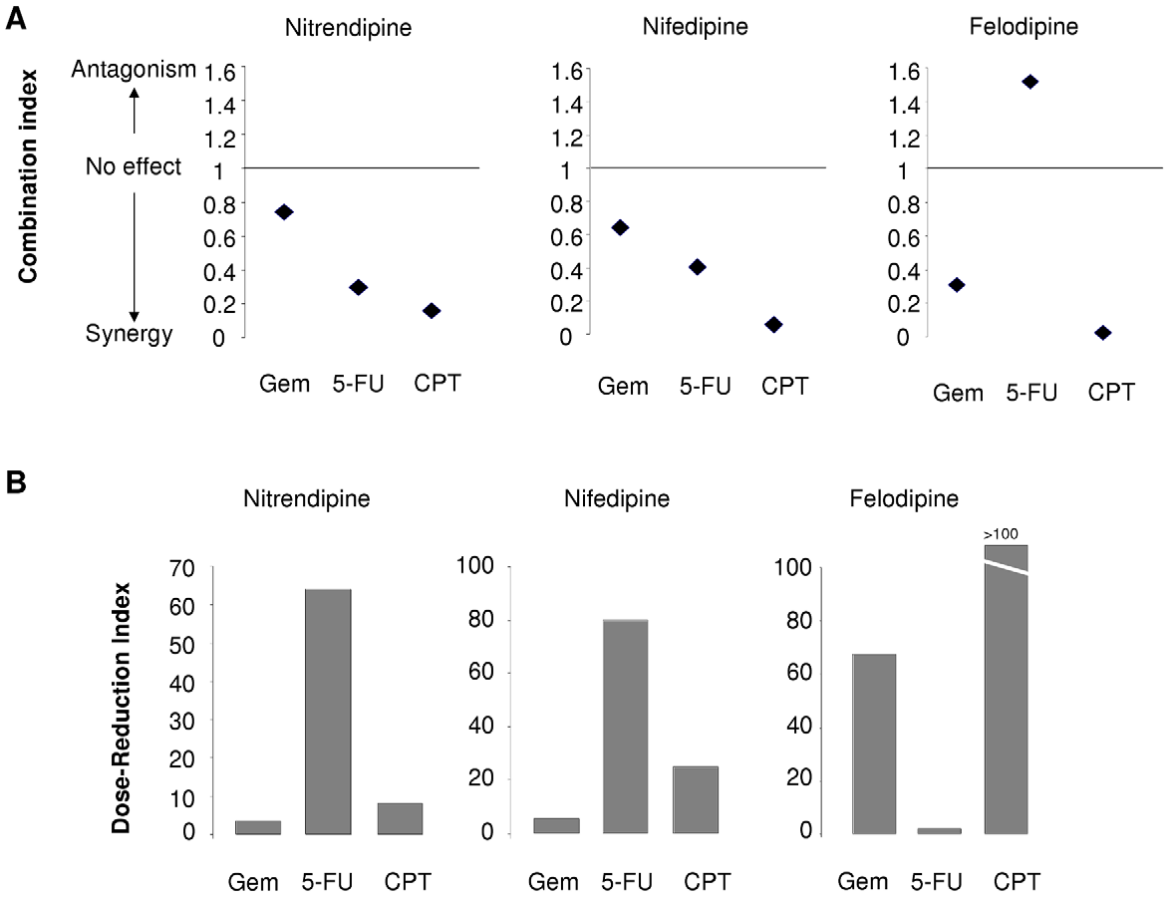
Synergistic interactions between candidate agents and chemotherapeutic agents *in vitro*. Panel A: Cytotoxicity was assessed in Mz-ChA-1 cells incubated with several different concentrations of nitrendipine, nifedipine or felodipine with gemcitabine (GEM), camptothecin (CPT) or 5-fluoro-uracil (5-FU) in a fixed ratio of concentrations. Potential interactions were evaluated using the median effects analysis of Chou and Talalay, and the combination index (CI) derived. A CI<0.8 or >1.2 was considered to represent a significant interaction, with a CI<1 indicating a synergistic interaction and a CI>1 indicating an antagonistic effect. At a fractional effect of 0.5, which corresponds to the IC50, a synergistic effect occurred between nifedipine or nitrendipine and all three chemotherapeutic agents studied. Synergism was also observed between felodipine and either gemcitabine or camptothecin However, an antagonistic interaction was observed between felodipine and 5-fluoro-uracil. Panel B: The impact of the candidate agents on dose-reduction of other chemotherapeutic agents when used in combination was determined from cytotoxicity studies of agents in combination at fixed molar concentrations and a dose-reduction index derived using the Calcusyn software at a fractional effect of 0.5. A dramatic effect in the dose-reduction index was observed with felodipine for gemcitabine and camptothecin, and for nifedipine and nitrendipine for 5-fluorouracil.

In order to evaluate the potential use of these compounds in the treatment of cholangiocarcinoma, we analyzed their interactions with selected conventional chemotherapeutic agents. The nature of the interactions between these compounds and gemcitibine, 5-fluorouracil, or camptothecin was evaluated using the median effects analysis of Chou and Talalay, and the combination index (CI) derived, with a CI<1 indicating a synergistic interaction. At a fractional effect of 0.5, a synergistic effect occurred between either camptothecin or gemcitabine and felodipine, nifedipine or nitrendipine in Mz-ChA-1 cells. While a synergistic effect was also observed between nitrendipine and nifedipine and 5-fluorouracil, analysis revealed an antagonistic effect between felodipine and 5-fluorouracil (Figure 4A). Similarly, a dramatic effect in the dose-reduction index was observed in combinations of felodipine with either gemcitabine or camptothecin (Figure 4B). These observations strongly suggest that these compounds may be of value in improving responses to chemotherapy when used in combination therapy. Furthermore, the divergent interactions with 5-fluorouracil emphasize the need for individual assessment of each agent.

### Effect of felodipine and gemcitabine *in vivo*

**Figure 5:**
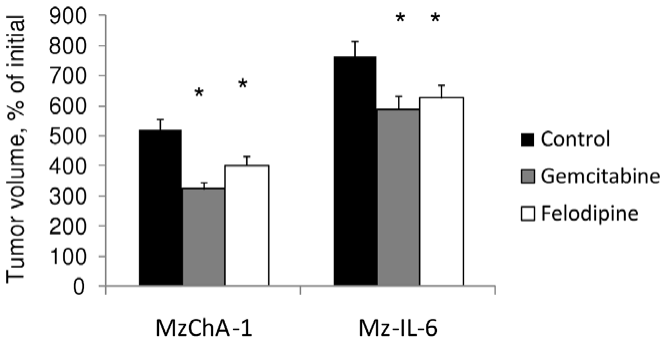
Felodipine reduces tumor cell xenograft growth. Panel A: Mz-ChA-1 and Mz-IL-6 tumor cell xenografts were established in nude mice. Once tumors were measureable, mice received gemcitabine 120 mg/kg or normal saline as a control every three days for 5 doses or felodipine 10 mg/kg once a week for two weeks. Tumor growth was evaluated by serial measurements. Data represent mean and standard error of normalized estimated tumor volume from 6 xenograft tumors for each cell type and for each group obtained on day 36 after subcutaneous implantation. * p<0.05 compared to respective control group.

**Figure 6:**
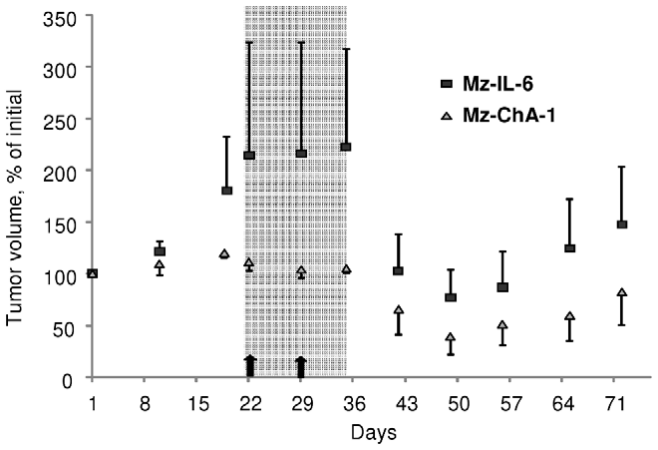
Effect of felodipine and gemcitabine on IL-6 expressing tumor cell xenograft growth. The effect of co-administration of felodipine and gemcitabine on Mz-ChA-1 and Mz-IL-6 tumor cell xenograft growth was assessed. The shaded region indicates the duration of treatment with gemcitabine, 120 mg/kg, every 3 days for 5 doses, whereas the solid arrows indicate the administration of felodipine. Tumor growth was markedly reduced when felodipine was given along with gemcitabine. Thus, felodipine can enhance the effects of gemcitabine on tumor cell growth *in vivo*. Data represent mean and standard error of normalized estimated tumor volume obtained using caliper measurements from 4 xenografts for each cell type.

Gemcitabine is used in clinical practice for biliary cancers. The greatest synergy with gemcitabine was observed with felodipine. Thus, we examined the *in vivo* effect of combining gemcitabine and felodipine. We used a subcutaneous tumor cell xenograft model and evaluated the response to treatment by serial measurements of tumor size. We started by analyzing the effects of either felodipine or gemcitabine on tumor cell xenograft growth. A reduction in rate of tumor growth was seen in Mz-ChA-1 and Mz-IL-6 xenografts with both agents (Figure 5). The effects of felodipine on tumor volume were less than those observed with gemcitabine alone making its use as a single agent unlikely. We also studied the effects of a combination of felodipine and gemcitabine on Mz-ChA-1 and Mz-IL-6 xenografts. Treatment consisted of i.p injections of gemcitabine 120 mg/kg every three days for a total of five doses, along with oral administration of 10 mg/kg felodipine once a week for two weeks. Consistent with predictions from our *in vitro* studies, a reduction in tumor cell growth was observed with both MzChA-1 and Mz-IL-6 xenografts (Figure 6).

### Effect of felodipine on gene expression

**Figure 7:**
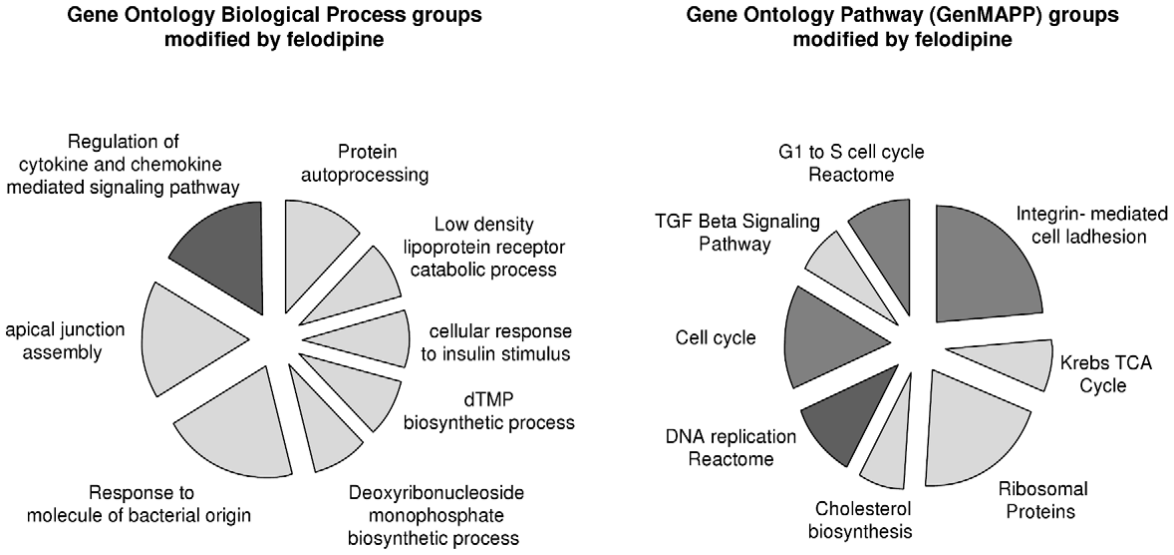
Gene ontology analysis of felodipine-induced gene expression. Mz-ChA-1 cells were incubated with 20 µM felodipine for 72 hours. RNA was then extracted and gene expression analysis was performed as indicated in the methods section. Gene Ontology (GO) analysis was performed by using the XRAY software. A set of differentially expressed genes were found to be significantly over-represented in groups of the Gene Ontology Biological Process and Pathway classes (p<0.01).

In order to assess the effect of felodipine on gene expression in cholangiocarcinoma cells we assessed gene expression profile in Mz-ChA-1 cells after treatment with felodipine 20 µM or diluent control. According to Gene Ontology analysis for biological processes felodipine significantly regulated cytokine and chemokine mediated signaling pathways in accordance with our previous data. The GenMAPP analysis by pathways revealed that felodpine can modulate cell cycle, DNA replication and cell adhesion (Figure 7), thereby supporting mechanisms by which felodipine could control cell proliferation and viability.

## Discussion

In this study, we show the utility of using phenotype-based signature profiling for the identification of new therapeutics. The power of this approach based on bioinformatics analysis of genome-scale information associated with a specific phenotype is that it does not require knowledge of gene-specific targets, nor does it require specific identified cellular targets involved in tumor growth. Thus, this is a powerful approach that may be useful to identify therapies that target a cancer phenotype that can be distinguished based on genomic expression.

The role of IL-6 as a biological determinant of cholangiocarcinoma growth has been demonstrated in both cell culture and tumor cell xenograft models [18], [26]–[28]. As such, targeting the production of IL-6, or the cellular signaling pathways activated by IL-6 is a rational approach to identifying new therapeutic targets. However, this pleiotropic cytokine can mediate several diverse cellular responses to injury or inflammation. Moreover, chronic cytokine stimulation can induce a wide range of cellular effects, via the activation of several different cell signaling pathways and altered expression of a broad range of target genes [29]. Thus, therapies targeting IL-6 are likely to have several undesirable effects. Rather than targeting a single gene or signaling pathway, we chose a systematic approach aimed at targeting the overall genomic response to chronic cytokine stimulation. The usefulness and validity of this unique approach is exemplified by the identification of a potent group of drugs with therapeutic potential for use in cholangiocarcinoma, a tumor type for which effective therapy is lacking.

There are several important implications of this work. The identification of a group of structurally related compounds with potential therapeutic efficacy is an unexpected observation. We are not aware of any reports of the evaluation or use of these compounds as systemic therapies for cholangiocarcinoma. Although these compounds are widely used in clinical practice, there is a lack of knowledge as to their effects on human cancers [30]. Previous concerns regarding the potential risk of increasing cancer have not been supported by epidemiological studies [31]. In contrast, some studies have alluded to a decrease in incidence of certain malignancies such as prostate or colon cancer amongst persons taking calcium channel blockers [32], [33]. However, the changes have been small and moreover the potential use of these drugs as therapeutic rather than preventive agents has not been well characterized. Given the widespread use of these agents for the treatment of cardiovascular conditions, it is unlikely that they will be exploited as therapeutics by themselves, but may have promise for use in combination therapy. Our data suggests that calcium channel blockers can inhibit tumor cell growth through a modulation of different pathways such as cytokine-mediated pathways, cell cycle and DNA repair. Recent evidence also shows that calcium channel blockers can reverse chemotherapy resistance by modulating Multidrug resistance (MDR)-related genes such asATP-binding cassette transporter B1 gene [34].

These observations warrant further study, and should generate hypotheses for laboratory, patient or population-based studies. First, laboratory studies to explore and identify the potential mechanisms by which these compounds exert their cytotoxicity in cholangiocarcinoma, effects in other cancer types, as well as broader studies of the role of calcium channel blockade in cancer cells are necessary. Next, clinical trials to evaluate the effect of concomitant calcium channel blockers on responses to treatment of cholangiocarcinoma should be considered. Finally, epidemiological studies to examine the effects of concomitant calcium channel blocker therapy on the outcomes of cancer treatment are also justified.

We chose to focus on members of a structurally related group of compounds because of their strong representation amongst compounds with a negative correlation. However, other attractive candidates for further study were also identified. An example is monastrol, a compound with a high negative correlation to the query signature. Monastrol can inhibit cell cycle progression and is being evaluated as a potential anticancer agent. Another example is geldanamycin, a heat shock protein inhibitor which has also shown promise in other cancers. These compounds could be considered for further experimental study of their potential for use in the treatment of cholangiocarcinoma.

In this study, we have demonstrated the use of a computational bioinformatics driven approach to drug discovery involving the identification of potential therapeutic drug candidates capable of targeting a specific phenotype. Although focused on cancer, this approach can be expanded to other disease phenotypes. Given the increasing public availability of genomic data, we predict that this approach to *in silico* drug discovery will be an attractive approach that could be fruitfully explored in many other disease phenotypes. Moreover, we predict that identification of novel agents based on this approach of genomic signature profiling will generate several new hypotheses, which in turn may enhance our understanding of biological processes. We speculate that broader use of this strategy will be useful to move us into the realm of phenotype-targeted therapy for a broad range of human conditions.

## Materials and Methods

### Cells and cell lines

Mz-ChA-1 cells (metastatic gall bladder cancer), TFK cells (common bile duct cancer), KMCH-1 cells (cholangiocellular-hepatocellular cancer), or CC-LP-1 (intrahepatic cholangiocarcinoma) were cultured as previously described [18], [26], [27]. Mz-ChA-1 cells were stably transfected with full-length IL-6 and clones that over-expressed IL-6 were selected and propagated (Mz-IL-6) as previously described [18]. PLC/PRF-5 and Hep-3B cells derived from hepatocellular cancer were cultured in DMEM with 10% fetal bovine serum (FBS), 1% L-glutamine, and 1% antimycotic-antibiotic mix.

### Bioinformatics analysis

Whole genome microarray analysis was performed using Affymetrix U133 plus 2 chips (Affymetrix, Santa Clara, CA) [17]. Data have been deposited in GEO (accession #: GSE11883). Genes with a 1.5 fold or greater difference in expression between controls and IL-6 over-expressing cells were selected. A gene signature associated with IL-6 over-expression was derived based on differentially expressed genes that were both significantly and consistently altered. Bioinformatics analysis consisted of querying this gene signature across the Connectivity Map data set (build01) which contains 453 Affymetrix gene expression signatures of 164 bio-active small-molecule perturbagens with diverse biological activities [35]. A "connectivity score" for each perturbagen was calculated based on the Kolmogorov-Smirnov statistic for up- or down-regulated genes. This score represents the correlation between the query signature and gene expression associated with a treatment-control pair, or instance, with negative scores correlating inversely with the query gene signature.

### Cell viability assays

Cells were seeded into 96-well plates at a cell density of 5×10^3^ per well in appropriate media with 5% FBS and incubated at 37°C with 5% CO2. Cells were then incubated with varying concentrations of felodipine, nitrendipine, nifedipine or the appropriate diluent (DMSO) control in 50 µl of media. At selected time points, cell viability was assessed using a colorimetric assay (CellTiter 96 Aqueous, Promega Crop., Madison, WI). The IC50 was calculated using the XLfit software (IDBS, Burlington, MA). For studies in calcium-free media, Mz-ChA-1 cells were cultured in calcium free media supplemented with L-glutamine (1%), fetal bovine serum (5%), and antibiotic/antimycotic (1%) in 96-well plates, and cell viability assessed after incubation of cells with felodipine, nitrendipine or nifedipine for 72 hrs.

### Apoptosis assays

Cells were plated overnight in a 4-well chamber slide (5×10^4^ cells per well) in 500 µl of media and incubated at 37°C with 5% CO2. Cells were then incubated with felodipine, nitrendipine and nifedipine and the extent of cell apoptosis was assessed after 24 hours. Cells with morphological changes indicative of cell death by apoptosis were quantitated using fluorescence microscopy after incubating with 4′, 6-diamidino-2-phenylindole (DAPI). For caspase activation assays, cells were prepared in a similar manner and caspase 3/7 activity assessed after 24 hours using a commercial luminometric assay (Caspase-Glo 3/7 assay, Promega Corp., Madison, WI).

### Transfection

Transfections were performed by nuclear transfection using the Nucleofector system solution T program T20 (Amaxa Biosystems, Koln, Germany). Cells (1–2×10^6^) were suspended in 100 µL Nucleofector solution (Amaxa Biosystems) containing 100 nM siRNA antiCACNG6 or control siRNA obtained from Dharmacon (Lafayette, CO). Transfected cells were then re-suspended in culture media containing 10% FBS for 48 hours before study.

### PCR analysis

RNA was extracted using the RNeasy mini kit (Qiagen, Valencia, CA). One µg RNA was reverse transcribed to cDNA in 20 µl reaction volume using 5x iScript ^™^ cDNA synthesis kit (BioRad, Hercules, CA). PCR performed using the following primers: left CACNG6 5′-*gagaatgcacgcatctttca*-3′ and right CACNG6 5′-*caactcggagcaggaactct*-3′.PCR parameters were as follows: 10 minutes at 95°C, and then 40 cycles of 15 seconds at 95°C, 1 minute at 53–60°C.

### Microarray analysis

RNA was extracted using the RNeasy mini kit (Qiagen, Valencia, CA), reverse transcribed and hybridized onto Affymetrix HG-U133_Plus_2 arrays (Affymetrix, Santa Clara, CA). Data were normalized with full quantile normalization, corrected for background and transformed by taking the base-2 Logarithm of 0 plus the probe score. Data have been deposited in GEO (accession #: GSE23427). For each gene, expression values were paired by probe-id and a paired t-test was used to identify differential gene expression between the groups with a Type I Error of 0.01. GO analysis was then performed XRAY (version 3.998) software (Biotique Systems Inc, Reno, NV).

### Drug synergy interactions

Interactions between several diverse chemotherapeutic agents (gemcitibine, 5-fluorouracil, or camptothecin) and candidate agents (nitrendipine, nifedipine or felodipine) were evaluated by assessing cell viability in response to combinations of the chemotherapeutic agents and candidate agents in fixed ratio of concentrations. The results were analyzed using the median effects analysis of Chou and Talalay [36], and the combination index (CI) derived using the Calcusyn software program (Biosoft, Cambridge, United Kingdom). A CI<1 indicates a synergistic interaction, whereas a CI>1 indicates an antagonistic effect. For our analysis, we assigned a CI of 1.0±0.2 as indicating the absence of a significant interaction.

### Tumor cell xenograft studies

Eight-week-old male athymic *nu/nu* mice were obtained from Charles River Laboratories (Wilmington, MA) and fed food and water *ad libitum*. The mice were maintained in accordance with the Institutional Animal Care and Use Committee procedures and guidelines. They were housed 3 or 4 per cage, and fluorescent light was controlled to provide alternate light and dark cycles of 12 hours each. Parental Mz-ChA-1 or IL-6 over-expressing Mz-IL-6 cells (5×10^6^ cells) were suspended in 0.25 mL of extracellular matrix gel, and the mixture was injected subcutaneously into the right and left flanks. Xenograft growth was monitored by serial caliper measurements once tumors were palpable. A two-week treatment course was initiated after tumors were palpable, and tumor growth was monitored for at least 28 days after completion of treatment course. Treatments included felodipine 10 mg/kg po once a week for 2 weeks, gemcitabine 120 mg/kg i.p every three days for a total of 5 doses or normal saline i.p.

### Statistical analysis

Results are expressed as mean ± standard deviation, unless indicated otherwise. Comparisons between groups were performed using the two-tailed Student's *t* test. For tumor xenograft studies, mean tumor volumes in each group were compared at each time point using a *t*-test. The rate of tumor growth in animals was determined from the slope of the tumor growth time curve fitted by linear regression, and the *F* test used to statistically compare slopes. Significance was accepted when *p* was less than 0.05.

### Chemicals and reagents

Gemcitabine was provided by Eli Lilly (Indianapolis, IN). Felodipine, nitrendipine, nifedipine, camptothecin, and 5-fluorouracil were obtained from Sigma-Aldrich, (St. Louis, MO), Verapamil was obtained from Calbiochem (San Diego, CA) and diluted in water. All reagents used were of the highest purity available.

## Supporting Information

Figure S1 **Cytotoxicity of felodipine and nitrendipine in hepatocellular cancer cells.** PLC/PRF-5 and Hep-3B cells were incubated with the candidate agents at the indicated concentrations. Cell viability was assessed after 72 hrs using a viable cell assay, and IC50 values were calculated after curve-fitting using the XLfit Software.
(TIF)

Figure S2 **Inhibition of CACNG6 reduces cell viability of malignant cholangiocytes**. Mz-IL-6 cells were transfected with siRNA anti-CACNG6 or siRNA control. Left panel: After 48 hours RNA was extracted and PCR was performed to assess CACNG6 mRNA expression. An image from a representative agarose gel of the PCR product is shown along with densitometric values of CACNG6/GAPDH expression. Right panel: After 48 hours of transfection cells were incubated with medium alone, DMSO or felodipine at 20 µM and cell viability was assessed after 72 hours as indicated in the methods section. *: p<0.05 vs control.
(TIF)

Table S1 **Gene signature used for bioinformatic analysis.** A genomic signature consisting of 43 differentially expressed genes associated with IL-6 over-expression was derived from whole genome analysis using Affymetrix U133 Plus 2.0 arrays. The fold change in gene expression in IL-6 over-expressing cells relative to controls is indicated.
(DOC)

Table S2 **Genes involved in calcium regulation modulated by IL-6.** Microarray analysis was performed using Affymetrix U133 plus 2 chips in Mz-ChA-1 and Mz-IL-6 cells. The ratio of the expression of selected genes involved in calcium regulation which were significantly expressed and present in all samples is shown.
(DOC)
