# Supplementary figures and images for: Targeting the IL-6 Dependent Phenotype Can Identify Novel Therapies for Cholangiocarcinoma
Source: PLoS One. 2010 Dec 16;5(12):e15195. doi: 10.1371/journal.pone.0015195 (PMC3002961; doi:10.1371/journal.pone.0015195)

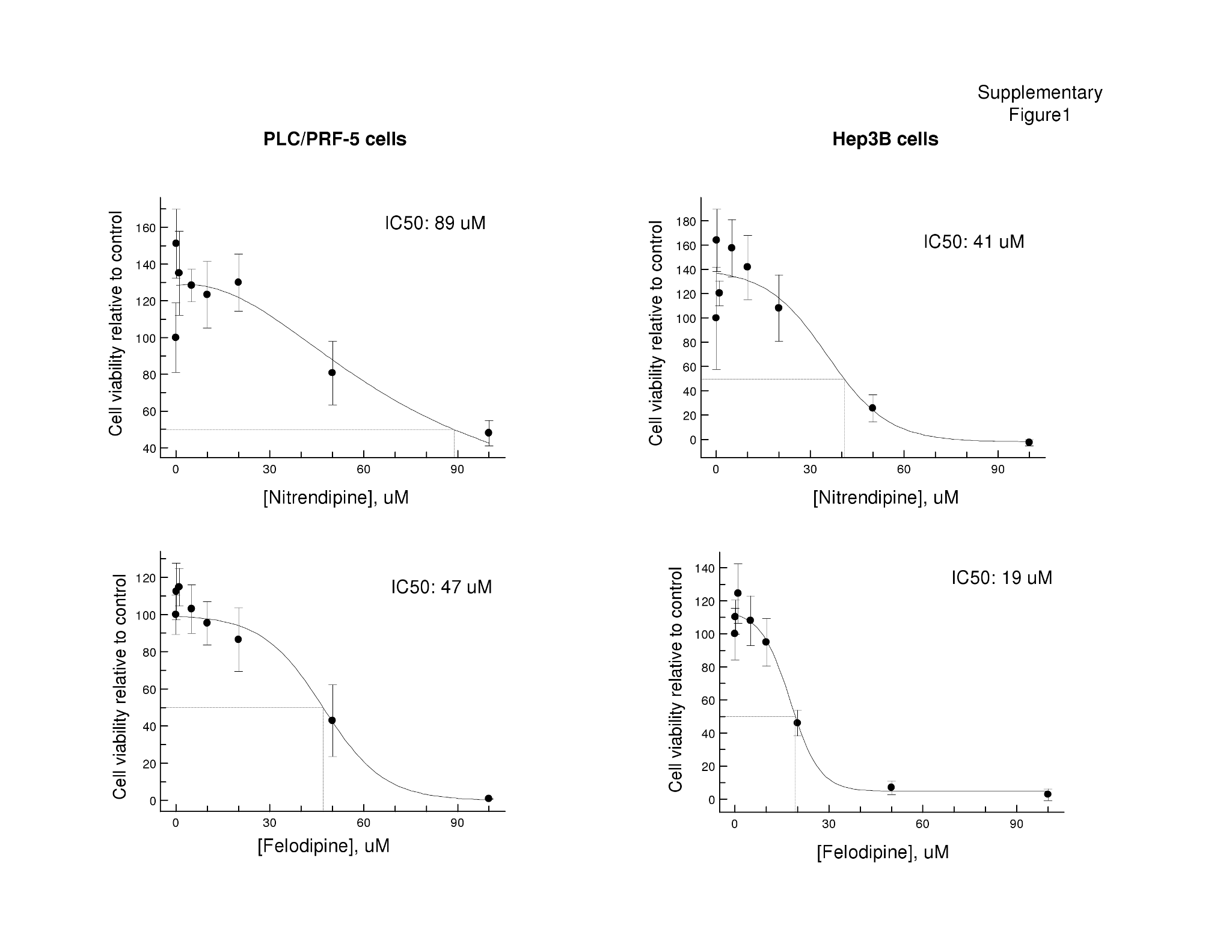

Supplement: Figure S1 — Cytotoxicity of felodipine and nitrendipine in hepatocellular cancer cells. PLC/PRF-5 and Hep-3B cells were incubated with the candidate agents at the indicated concentrations. Cell viability was assessed after 72 hrs using a viable cell assay, and IC50 values were calculated after curve-fitting using the XLfit Software. (TIF) [file pone.0015195.s001.tif]

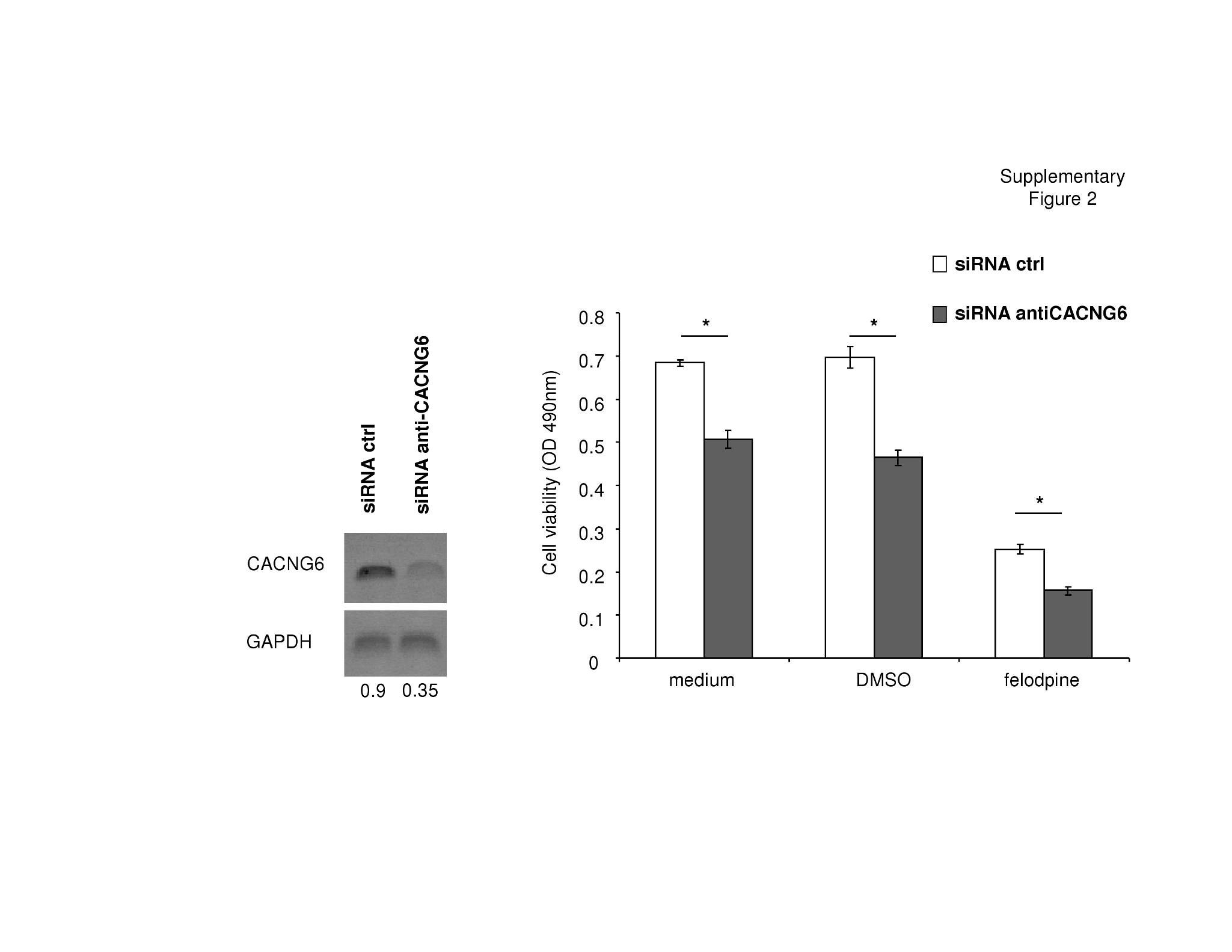

Supplement: Figure S2 — Inhibition of CACNG6 reduces cell viability of malignant cholangiocytes. Mz-IL-6 cells were transfected with siRNA anti-CACNG6 or siRNA control. Left panel: After 48 hours RNA was extracted and PCR was performed to assess CACNG6 mRNA expression. An image from a representative agarose gel of the PCR product is shown along with densitometric values of CACNG6/GAPDH expression. Right panel: After 48 hours of transfection cells were incubated with medium alone, DMSO or felodipine at 20 µM and cell viability was assessed after 72 hours as indicated in the methods section. *: p<0.05 vs control. (TIF) [file pone.0015195.s002.tif]
