# Supplementary material for: Targeting the IL-6 Dependent Phenotype Can Identify Novel Therapies for Cholangiocarcinoma
Source: PLoS One. 2010 Dec 16;5(12):e15195. doi: 10.1371/journal.pone.0015195 (PMC3002961; doi:10.1371/journal.pone.0015195)
Supplement: Table S1 — Gene signature used for bioinformatic analysis. A genomic signature consisting of 43 differentially expressed genes associated with IL-6 over-expression was derived from whole genome analysis using Affymetrix U133 Plus 2.0 arrays. The fold change in gene expression in IL-6 over-expressing cells relative to controls is indicated. (DOC) [file pone.0015195.s003.doc]

| Gene | Gene title | Fold-change |
| --- | --- | --- |
| IGF1R | insulin-like growth factor 1 receptor | -1.5 |
| SCD | stearoyl-CoA desaturase | -1.5 |
| OAS1 | 2',5'-oligoadenylate synthetase 1 | -1.5 |
| NEDD9 | neural precursor cell expressed | -1.5 |
| AKR1C4 | aldo-keto reductase family 1, member C4 | -1.5 |
| RARRES3 | Retinoic acid receptor responder 3 | -1.6 |
| CPE | carboxypeptidase E | -1.6 |
| IFI27 | interferon, alpha-inducible protein 27 | -1.6 |
| IGFBP3 | insulin-like growth factor binding protein 3 | -1.6 |
| ISG15 | ISG15 ubiquitin-like modifier | -1.6 |
| CCL5 | chemokine (C-C motif) ligand 5 | -1.7 |
| BMP2 | bone morphogenetic protein 2 | -1.7 |
| SCD | stearoyl-CoA desaturase (delta-9-desaturase) | -1.8 |
| CST2 | Cystatin SA | -1.8 |
| TESC | Tescalcin | -1.8 |
| ALDH3A1 | aldehyde dehydrogenase 3 family, memberA1 | -1.8 |
| IFIT1 | interferon-induced protein with tetratricopeptide repeats 1 | -1.8 |
| BMP2 | bone morphogenetic protein 2 | -1.8 |
| BACE2 | beta-site APP-cleaving enzyme 2 | -1.9 |
| PRSS23 | protease, serine, 23 | -1.9 |
| STEAP4 | STEAP family member 4 | -1.9 |
| S100A4 | S100 calcium binding protein A4 | -1.9 |
| CEACAM5 | carcinoembryonic antigen-related cell adhesion molecule | -2.0 |
| MUC1 | mucin 1, cell surface associated | -2.1 |
| PYCRL | pyrroline-5-carboxylate reductase-like | -2.2 |
| MUC5AC | mucin 5AC, oligomeric mucus/gel-forming | -2.2 |
| TAPBPL | TAP binding protein-like | -2.4 |
| PLA2G4A | phospholipase A2, group IVA | -2.5 |
| PSCA | prostate stem cell antigen | -2.5 |
| KRT81 | keratin 81 | -4.5 |
| PFTK1 | PFTAIRE protein kinase 1 | 1.5 |
| VIL1 | villin 1 | 1.5 |
| STMN3 | stathmin-like 3 | 1.5 |
| CEP350 | centrosomal protein 350kDa | 1.6 |
| IGF2 | insulin-like growth factor 2 | 1.6 |
| USP34 | ubiquitin specific peptidase 34 | 1.8 |
| LPHN2 | latrophilin 2 | 1.9 |
| DIS3 | DIS3 mitotic control homolog | 2.0 |
| AK2 | adenylate kinase 2 | 2.0 |
| HLA-DRB1 | major histocompatibility complex, class II, DR beta 1 | 2.6 |
| MTM1 | myotubularin 1 | 2.9 |
| CD96 | CD96 molecule | 3.1 |
| MBNL2 | muscleblind-like 2 | 4.3 |
