# Supplementary material for: Targeting the IL-6 Dependent Phenotype Can Identify Novel Therapies for Cholangiocarcinoma
Source: PLoS One. 2010 Dec 16;5(12):e15195. doi: 10.1371/journal.pone.0015195 (PMC3002961; doi:10.1371/journal.pone.0015195)
Supplement: Table S2 — Genes involved in calcium regulation modulated by IL-6. Microarray analysis was performed using Affymetrix U133 plus 2 chips in Mz-ChA-1 and Mz-IL-6 cells. The ratio of the expression of selected genes involved in calcium regulation which were significantly expressed and present in all samples is shown. (DOC) [file pone.0015195.s004.doc]

| Gene Name | Title | Mz-IL-6 / Mz-ChA-1 |
| --- | --- | --- |
| ITPR2 | inositol 1,4,5-triphosphate receptor, type 2 | 0.94 |
| ITPR3 | inositol 1,4,5-triphosphate receptor, type 3 | 0.70 |
| ITPR3 | inositol 1,4,5-triphosphate receptor, type 3 | 1.40 |
| ATP2B1 | ATPase, Ca++ transporting, plasma membrane 1 | 1.18 |
| ATP2B1 | ATPase, Ca++ transporting, plasma membrane 1 | 1.20 |
| ATP2B3 | ATPase, Ca++ transporting, plasma membrane 3 | 0.65 |
| ATP2B4 | ATPase, Ca++ transporting, plasma membrane 4 | 0.98 |
| ATP2B4 | ATPase, Ca++ transporting, plasma membrane 4 | 1.09 |
| ATP2B4 | ATPase, Ca++ transporting, plasma membrane 4 | 0.66 |
| TRPV3 | transient receptor potential cation channel,subfamily V, member 3 | 0.69 |
| CACNA1F | L-type calcium channel alpha-1 subunit | 0.82 |
| CACNA1A | calcium channel, voltage-dependent, PQ type, alpha 1A subunit | 1.13 |
| CACNG5 | calcium channel, voltage-dependent, gamma subunit 5 | 1.51 |
| CACNG6 | calcium channel, voltage-dependent, gamma subunit 6 | 1.80 |
